# Supplementary material for: Resolving the full spectrum of human genome variation using Linked-Reads
Source: Genome Res. 2019 Apr;29(4):635–45. doi: 10.1101/gr.234443.118 (PMC6442396; doi:10.1101/gr.234443.118)
Supplement: Supplemental Material [file supp_gr.234443.118_Supplemental_longranger-2.2.zip › longranger-2.2.tar.bz2!/lr2.2-public/lib/stan/cmdstan/stan/doc/api/html/structvalidate__identifier.html]

Stan: validate\_identifier Struct Reference


|  |  |
| --- | --- |
|  | Stan  2.5.0  probability, sampling & optimization |


- Main Page
- Related Pages
- Namespaces
- Classes
- Files

- Class List
- Class Index
- Class Hierarchy
- Class Members

All Classes Namespaces Files Functions Variables Typedefs Enumerations Enumerator Friends Macros Pages

Classes |
Public Member Functions |
Static Public Member Functions |
Public Attributes |
List of all members

validate\_identifier Struct Reference

`#include <var_decls_grammar_def.hpp>`

|  |  |
| --- | --- |
| Classes | |
| struct | result |
|  | |

|  |  |
| --- | --- |
| Public Member Functions | |
| void | reserve (const std::string &w) |
|  | |
| bool | identifier\_exists (const std::string &identifier) const |
|  | |
|  | validate\_identifier () |
|  | |
| bool | operator() (const std::string &identifier, std::stringstream &error\_msgs) const |
|  | |

|  |  |
| --- | --- |
| Static Public Member Functions | |
| template<typename S , typename T > | |
| static bool | contains (const S &s, const T &x) |
|  | |

|  |  |
| --- | --- |
| Public Attributes | |
| std::set< std::string > | reserved\_word\_set\_ |
|  | |
| std::set< std::string > | const\_fun\_name\_set\_ |
|  | |

## Detailed Description

Definition at line 305 of file var\_decls\_grammar\_def.hpp.

## Constructor & Destructor Documentation

|  |  |  |  |  |
| --- | --- | --- | --- | --- |
| validate\_identifier::validate\_identifier | ( |  | ) |  |

inline

Definition at line 328 of file var\_decls\_grammar\_def.hpp.

## Member Function Documentation

template<typename S , typename T >

|  |  |  |  |
| --- | --- | --- | --- |
| static bool validate\_identifier::contains | ( | const S & | *s*, |
|  |  | const T & | *x* |
|  | ) |  |  |

inlinestatic

Definition at line 317 of file var\_decls\_grammar\_def.hpp.

|  |  |  |  |  |  |
| --- | --- | --- | --- | --- | --- |
| bool validate\_identifier::identifier\_exists | ( | const std::string & | *identifier* | ) | const |

inline

Definition at line 322 of file var\_decls\_grammar\_def.hpp.

|  |  |  |  |
| --- | --- | --- | --- |
| bool validate\_identifier::operator() | ( | const std::string & | *identifier*, |
|  |  | std::stringstream & | *error\_msgs* |
|  | ) |  | const |

inline

Definition at line 474 of file var\_decls\_grammar\_def.hpp.

|  |  |  |  |  |  |
| --- | --- | --- | --- | --- | --- |
| void validate\_identifier::reserve | ( | const std::string & | *w* | ) |  |

inline

Definition at line 312 of file var\_decls\_grammar\_def.hpp.

## Member Data Documentation

std::set<std::string> validate\_identifier::const\_fun\_name\_set\_

Definition at line 307 of file var\_decls\_grammar\_def.hpp.

std::set<std::string> validate\_identifier::reserved\_word\_set\_

Definition at line 306 of file var\_decls\_grammar\_def.hpp.

---

The documentation for this struct was generated from the following file:

- src/stan/gm/grammars/var\_decls\_grammar\_def.hpp


---

[ Stan Home Page ]

*© 2011–2014,
Stan Development Team.*
